# The Originality of Neuro Rehabilitation Protocols in a Definitive Case of Syringomyelia Related to Chiari I Malformation

**DOI:** 10.7759/cureus.45157

**Published:** 2023-09-13

**Authors:** Pooja R Tiwari, Ruchika J Zade, Snehal S Samal

**Affiliations:** 1 Neuro Physiotherapy, Ravi Nair Physiotherapy College, Wardha, IND

**Keywords:** syringomyelia, physiotherapy, neurological symptoms, cm1, chiari malformation

## Abstract

Syringomyelia is a center-medullary syndrome characterized by the presence of fluid-filled spaces known as syrinx within the spinal canal. Arnold Chiari Malformation (CM-I), a rhombencephalon anomaly formerly identified as hindbrain hernia, is usually associated with it. This disorder causes the brain (cerebellum) to bulge through the opening in the skull known as the foramen magnum. Some asymptomatic patients may develop symptoms quickly if they jolt their heads and cough for a lengthy period of time. Syringomyelia can be caused by trauma, illness, inflammation, or previous surgery that affects the circulation of cerebral spinal fluid resulting in CSF flow obstruction. The discomfort is acute and progressive, radiating to the neck and shoulder, and is accompanied by sensory loss, motor atrophy, decreased hearing, oscillopsia, and cerebellar abnormalities. This case report is of a 39-year-old woman diagnosed with syringomyelia associated with Arnold Chiari malformation and showed similar symptoms managed by foramen decompression and tonsillar elevation surgery. It involves removing a small piece of bone from the skull and a small section of the 1st vertebra from the back of the neck and head. In this way, there is an increase in skull space. Decompression of the spinal canal increases the size of the subarachnoid cisterns and constricts the syrinx cavity. After surgery, physiotherapy was advised because all superficial sensations over C8 and T1 were diminished, the range of motion along with strength was reduced, doing daily activities was difficult, and quality of life was affected. So, by decreasing symptoms and improving the patient's quality of life, physiotherapy improved the patient's condition significantly in this case report. The rationale of this study is to show the importance of physiotherapy in recovering after a neurological condition followed by corrective neurosurgery.

## Introduction

Syringomyelia is typically conjugated with Arnold Chiari Malformation (CM-I) and is unusual, having stages of instability and progression in the clinical course that can last months or years. The natural history of this condition is still not completely understood by professionals but it may be indicative of a sudden onset of symptoms in a previously asymptomatic patient if they suddenly jolt their heads and cough for a prolonged period probably due to the increased descent of tonsils. Up to 5% of paraplegia cases are caused by syringomyelia [1]. A partial restriction of cerebrospinal fluid (CSF) flow may occur in the spinal subarachnoid space due to other pathophysiological factors as a result of trauma, infection, inflammation, and previous surgery, syringomyelia may develop. Mechanical arachnoid scarring is connected with previous surgery which disturbs the circulation of cerebral spinal fluid within the central canal of the spinal cord which further leads to congestion of CSF flow [2]. Syringomyelia shows a close association with Chiari malformation which is an abnormality where the brain (cerebellum) protrudes through an opening that is the foramen magnum of the skull to the spinal canal. This can occur in between the second to fifth decade of life with a prevalence of 8.4/100,000 or 2% with a geographic and ethnic variation [3,4]. The condition manifests clinical signs due to the presence of longitudinal cysts in the cervicothoracic and cervical regions [4]. Coughing (Valsalva maneuvers) can produce a rapid increase in intracranial CSF volume in patients with CM1, resulting in short-term headaches and neck pain in the suboccipital region. The pain is rapid and progressive, radiating vertex behind the eyes, inferiorly to the neck and shoulder, sensory loss, and motor atrophy. Oto-neurologic symptoms include dysphagia caused by lower cranial nerve compression, reduced hearing, ear pressure, and oscillopsia. Cerebellar abnormalities include tremors, balance issues, and gait ataxia [5]. The condition can be managed through surgery followed by physiotherapy, even though the corrective surgery tries to restore CSF flow, numerous research studies have shown that there is a risk of severe and progressive neurological problems after the surgery where the physiotherapy aims to deal with a neurological problem. Physical therapy will not reduce the size of the cervical syrinx, but it can relieve the patient's symptoms and improve daily activities through a postural and biomechanical correction at the segmental level, culminating in normalization of cervical lordosis and thoracic kyphosis and significantly reduced tensional and compressive stress and strain on spinal cord parenchyma [6]. The study's rationale is that when patients have neurological conditions that are managed by corrective surgery but show weakness, continuous pain, and sensory and motor loss at some dermatomes and myotomes, physiotherapy is recommended for patients and plays an important role in rehabilitation as it can help with pain, decreased range of motion in the cervical and upper limbs, decreased power, loss of pain, touch, and temperature sensations, loss of coordination, altered gait cycle, and many others. By including patients in neurorehabilitation, the study aims to achieve pain management, quality enhancement, and faster recovery.

## Case presentation

**Figure 1:**
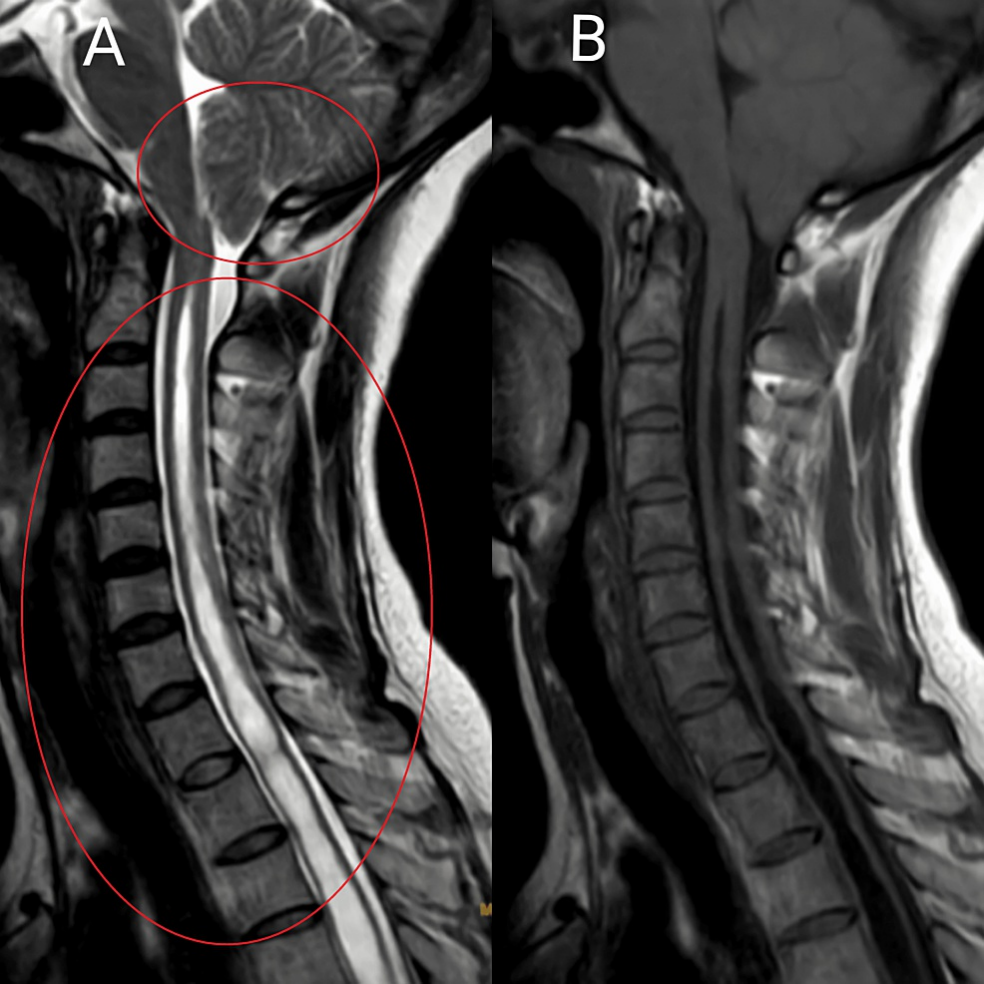
MRI of the cervical spine shows Arnold Chiari I malformation and syrinx formation from C2- D10 vertebral levels.

**Figure 2:**
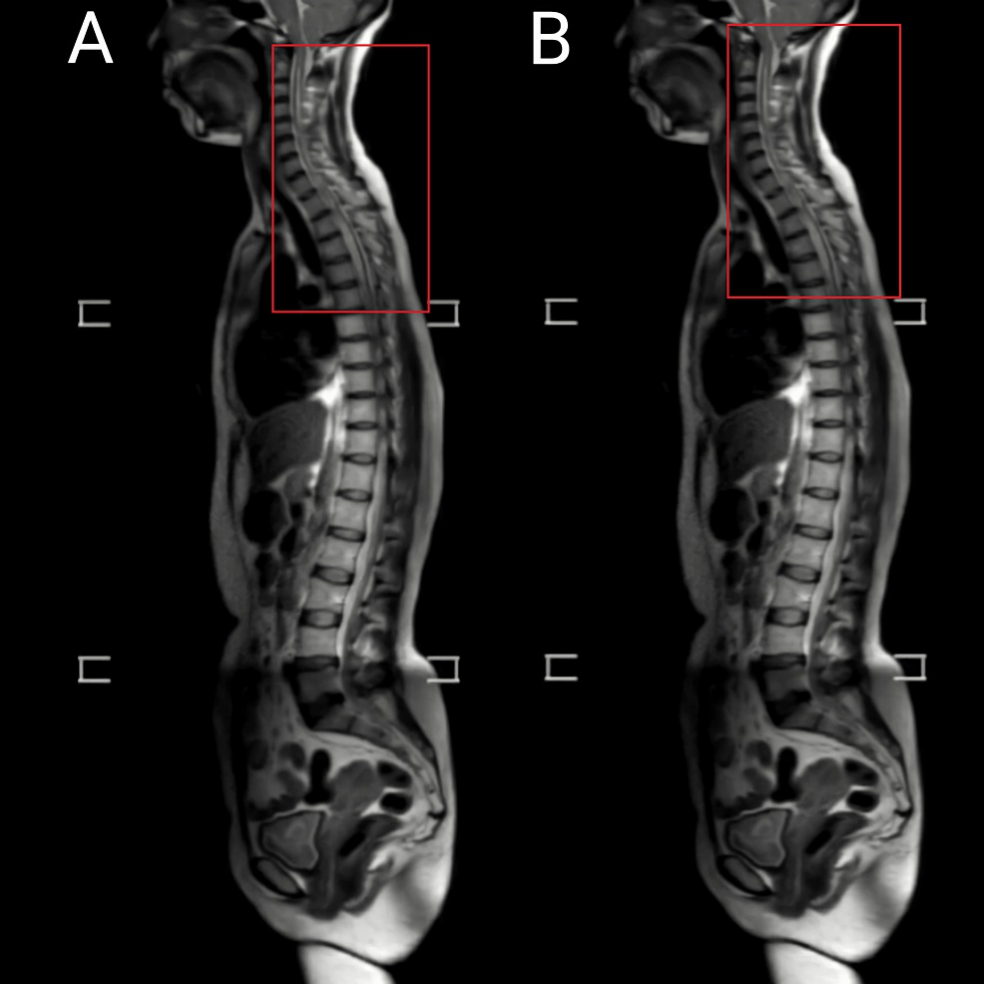
MRI shows intramedullary T2WI/STIR hyperintensity noted in spinal cord from C2- D10 vertebral levels suggestive of syrinx

A 39-year-old woman, a farmer by occupation came to the hospital with chief complaints of neck and upper back pain, tingling sensation, and weakness in bilateral upper limbs for one year. The pain was of insidious onset and gradually progressive. Four months later, the patient had difficulty holding objects, and the symptoms aggravated. The patient had no history of trauma, falls, or heavy weight lifting. The patient went to a private hospital and was advised to take an x-ray of the cervical spine after which the doctor gave her analgesic for pain but no improvement was observed after a month she came to the hospital with the above history and was suggested to undergo magnetic resonance imaging (MRI) of the cervical spine is shown in Figures 1, 2. MRI of the cervical spine shows Arnold Chiari I malformation and syrinx formation from C2- D10 vertebral levels and intramedullary T2WI/STIR hyperintensity noted in the spinal cord from C2- D10 vertebral levels suggestive of syrinx. So, doctors advised her her foramen magnum decompression with tonsillar elevation surgery. The patient was under general anesthesia the skin was incised paraspinal muscle was distracted and retracted, the C1 arch with occipital bone exposed magnum dura mater was opened in Y shape, and arachnoid adhesion cleared decompression of tonsil was done, duraplasty was done in lase manner using C2 patch and hemostasis done lastly the closure was done in four layers. Postoperatively she was kept in neuro-ICU for one day just for observation and was managed with analgesics, antibiotics, antiepileptic, and other supportive measures. Then she was shifted to the neurosurgery ward and referred to physiotherapy for further management. 

Pre-operative clinical findings

The patient was conscious, cooperative, and oriented to time, place, and person and was able to follow the commands. The patient complained of pain in the neck and upper back which was 8/10 according to the numeric pain rating scale (NPRS). On observation, posture was assessed in a sitting position from a lateral view the forward neck and kyphosis were observed. On examination, the superficial sensation in both upper limbs was diminished. The tone was normal according to the tone grading scale (TGS), reduced range of motion of the cervical spine and both upper limbs, and reduced strength.

Post-operative clinical findings

The physical therapist paid the patient a visit to the neurosurgery ward. The patient was seen in a supine lying position conscious, cooperative, and well-oriented to time, place, and, person. The pain at the suture site was 7/10 according to the numeric pain rating scale (NPRS). On observation, both the upper and lower limbs were extended and both ankles were planter flexed, the posture was assessed in a standing position through a lateral view revealing forward neck and kyphosis. While walking no heel strike, reduced hip and knee flexion reduced step length, and stride length were observed. On examination, the superficial sensations in the left upper limb including temperature and pain were diminished at C8 and T1 levels and the motor tone was normal that is +2 according to the tone grading scale (TGS) then cervical, upper limbs, and lower limbs range of motion was taken and are detailed in Tables 1, 2, the manual muscle testing was assessed and detailed in Table 3, and the coordination test is detailed in Tables 4, 5. The upper limb tension test for the ulnar nerve in the left upper limb was positive the and Romberg test was positive. 

**Table 1:** Cervical range of motion

Cervical Range of motion	Pre-interventional	Post-interventional
Flexion	34˚	45˚
Extension	35˚	45˚
Side Flexion	30˚	40˚
Rotation	55˚	70˚

**Table 2:** Upper limb and lower limb range of motion

Upper limb Range of motion	Pre– interventional Right	Post– interventional Right	Pre– interventional Left	Post– Interventional Left
Shoulder-Flexion	100˚	120˚	90˚	120˚
Extension	30˚	40˚	20˚	35˚
Abduction	90˚	110˚	90˚	110˚
Elbow- Flexion	0-140˚	0-150˚	0-140˚	0-150˚
Extension	140-0˚	150-0˚	140-0˚	150-0˚
Wrist Flexion	60˚	80˚	50˚	70˚
Extension	50˚	70˚	50˚	60˚
Radial deviation	10˚	20˚	10˚	20˚
Ulnar deviation	25˚	30˚	20˚	30˚
Hip Flexion	90˚	110˚	90˚	110˚
Extension	15˚	20˚	15˚	20˚
Abduction	35˚	40˚	35˚	40˚
Knee Flexion	100˚	120˚	100˚	120˚
Extension	100-0˚	120-0˚	100-0˚	120-0˚
Ankle planterflexion	45˚	50˚	45˚	50˚
Ankle Dorsiflexion	15˚	20˚	15˚	20˚

**Table 3:** Manual muscle testing for upper limb and lower limb

Manual muscle testing for upper limb	Pre-interventional Right	Post– interventional Right	Pre– interventional Left	Post– Interventional Left
Shoulder flexors	3/5	4/5	3/5	4/5
Extensors	3/5	4/5	3/5	4/5
Abductors	3/5	4/5	3/5	4/5
Elbow flexors	3/5	4/5	3/5	4/5
Extensors	3/5	4/5	2/5	4/5
Wrist flexors	3/5	4/5	2/5	3/5
Extensors	3/5	4/5	2/5	3/5
Hip Flexors	3/5	4/5	3/5	4/5
Hip Extensors	3/5	4/5	3/5	4/5
Hip Abductors	3/5	4/5	3/5	4/5
Knee Flexors	3/5	4/5	3/5	4/5
Knee Extensors	3/5	4/5	3/5	4/5
Ankle Dorsiflexors	3/5	4/5	3/5	4/5
Ankle Planterflexors	3/5	4/5	3/5	4/5

**Table 4:** Non-Equilibrium test

Non-equilibrium test	Pre– interventional Right	Post– interventional Right	Pre– interventional Left	Post– Interventional Left
Finger to finger	3	4	2	3
Finger to nose	3	4	2	3
Finger to therapist’s finger	3	4	2	3
Rebound test	Poor	Fair	Poor	Fair
Pronation supination	3	4	3	4
Mass grasp	2	4	2	3
Heel to shin	3	4	3	4

**Table 5:** Equilibrium test

Equilibrium test	Pre-interventional Eyes open	Post-interventional Eyes open	Pre-interventional Eyes close	Post-interventional Eyes close
Normal standing	Fair	Good	Fair	Good
Standing with narrow BOS	Fair	Good	Poor	Good
Standing with wide BOS	Fair	Good	Fair	Good
Tandem walking	Fair	Good	Poor	Fair
Standing on one leg	Poor	Good	Poor	Good

**Figure 3:**
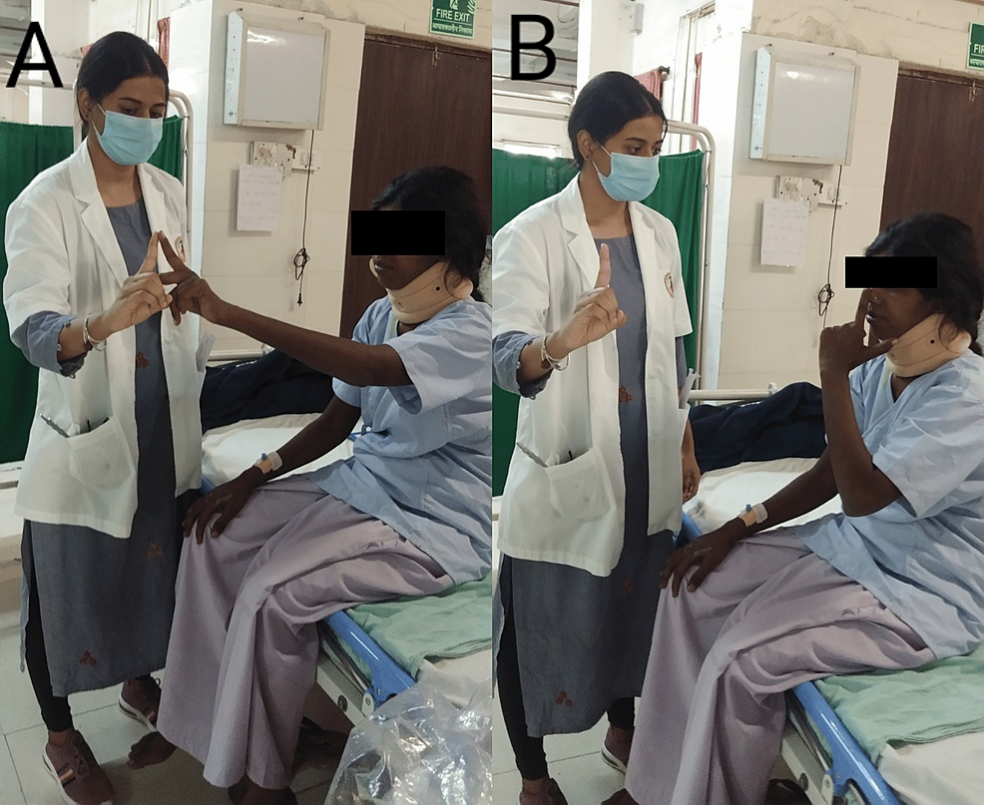
The non-equilibrium coordination exercise in both A & B therapist’s finger to nose

**Figure 4:**
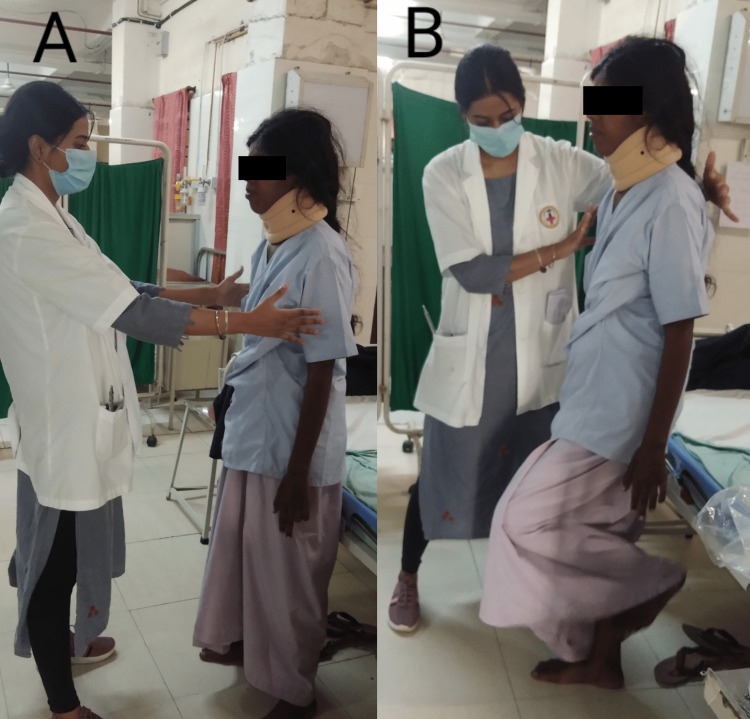
The equilibrium coordination exercises A. normal standing with a wide base of support and eyes closed, B. single leg stand

After performing all diagnostic assessments, the physiotherapist planned weekly protocols for the patient’s better recovery after surgery which is detailed in Table 6 given below. Figures 3, 4 explain the progression of physiotherapy protocols, as the patient was able to do both non-equilibrium and equilibrium exercises under the supervision of the therapist.

**Table 6:** Physiotherapy management

Days/Weeks	Goals	Physiotherapy Interventions	Dosage
Week 1	Patient education, relieving pain, preventing further complications after surgery, and maintaining all ranges of motion of the joints.	-Explaining the patient about the condition and counseling the family. -TENS and Cryotherapy were used to relieve pain. -The positioning was given in every 2 hours and the side-lying position was given to relieve pressure and prevent a further complications. -Active assisted range of motion to all joints	Cryotherapy was given 2-3 times a day for 10-15 minutes. Actively assisted ROM 10 reps thrice a day.
Week 2	-To improve transitions and bed mobility, -To improve range of motion and strength	-Mat exercise that is turning to each side and rolling. -For active range of motion ankle toe movement(B/L), heel slide (B/L), wrist elbow shoulder ROMs exercises (B/L) -fine movements exercise. (peg board, gel ball, and therapeutic clay or putty) -Progressive resisted exercises were started and progression was made by ½ kg weight every week.	5 reps × 2 sets, 10 reps × 3 sets
Week 3	-To improve trunk stability and correct the posture	-Weight transfer exercises were taught, Sit without support for 2min -Reach out in sitting with upper limb mobility and static back with Scapular sets and Shoulder shrugging.	10 reps × 2 sets with 5 seconds hold, 20 reps × 1 set
Week 4	-To improve balance and coordination, - Gait training -Home exercises program	- Finger to therapist’s finger shown in Figure 3, - Heel to the shin, -Frenkle exercise in supine lying -Frenkle’s exercise in sitting, -Sit to stand, -Weight shifting in standing, -standing with wide to narrow BOS and single stand shown in Figure 4, -reach out in standing. -Gait training activities in parallel bar -Ambulation with assistive device. -The taught exercises should be continued after discharge. Ergonomic advice was also given.	20 reps × 2 sets

**Figure 5:**
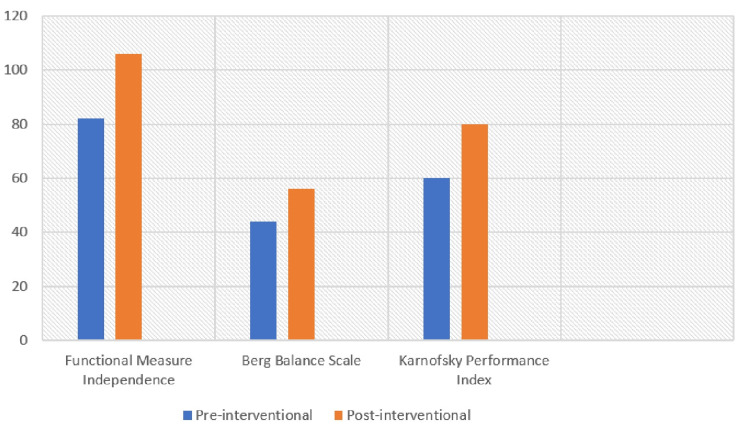
The outcome measures such as functional measures independence (FIM) from 82 to 106, Berg balance scale from 44 to 56, and Karnofsky performance index from 60 to 80 showed a significant improvement in patient independence by improving quality of life physiotherapeutic interventions.

This case report emphasizes physical therapy as a crucial component in a patient's overall recovery process, including pain management, enhanced range of motion and muscle strength, continued improved balance and coordination, solving complex gait impairments, and modifications in daily activities. The other outcome measures taken for the case showed positive results after surgery which is detailed in Table 7, and the results of outcome measures are explained graphically in Figure 5.

**Table 7:** Outcome measures

Outcome measures	Pre-interventional	Post-interventional
Functional Measure Independence	82	106
Berg balance scale	44	56
Karnofsky performance Index	60	80

## Discussion

Physiotherapy can't minimize the size of the cervical syrinx, but it may reduce symptoms and enhance daily activities through postural and biomechanical corrections at the segmental level, which leads to the normality of cervical lordosis and thoracic kyphosis and reduces tensional and compressive stress and strain on spinal cord tissue. Additional study on the impact of physiotherapy and biomechanical correction on the therapy of syringomyelia is required [7].

In this case study the patient underwent foramen decompression surgery with the main aim of constricting the syrinx cavity and creating significantly larger subarachnoid cisterns, Spinal decompression is intended to create sufficient subarachnoid cisterns. This is achieved by surgically reducing the cerebellar tonsils in conjunction with duraplasty, which enhances the clinical outcome for patients with CM. A sedentary lifestyle increases the risk of headaches in patients with CM, according to an incidental observation of the study [8]. Physiotherapy plays an important role in treating a variety of complaints that occur after a patient has received surgery, with pain being one of the most common problems. The study done by Salwa et al. concluded the use of infrared radiation and transcutaneous electrical nerve stimulation (TENS) as pain management physiotherapy interventions in postoperative syringomyelia patients results showed only a significant reduction in pain, as indicated by a reduction in the visual descriptive scale (VDS) pain score from 7/10 to 0/10. Despite this, the patient still complained about discomfort the following day, so the decrease in silent pain is only temporary [4]. The study done for similar neurological conditions in 2018 concluded that physiotherapy was effective in controlling pain and improving quality of life (QoL). The physiotherapy rehabilitation consisted of six sessions of myofascial release with dry needling, followed by vertebral adjustments with chiropractic techniques and specific muscle strengthening exercises after which the pain was relieved [9]. As confirmed by the diagnosis of a case of syringomyelia related to Chiari I malformation with tonsillar herniation in a 39-year-old female, this case study underlines the necessity of early assessment and timely referral of patients with unusual symptoms and neurological impairments. The example emphasizes the importance of imaging, particularly MRI, in the diagnosis process, as well as the importance of a collaborative approach to patient care. Clinicians, especially chiropractors, should maintain a high index of concern for significant underlying conditions and collaborate with other healthcare experts for proper assessment and treatment to enhance patient satisfaction [10]. However, Chu et al. 2022 found that an approach of conservative chiropractic and rehabilitative therapy benefited patients with persistent neck pain and headache worsened by a chronic, persistent cervicothoracic cord syrinx after foramen magnum decompression (FMD) for CM-I. Despite the effectiveness, the therapies utilized for patients with neck discomfort, headache, or other symptoms, and syringomyelia following FMD for CM-I continue to have limited and low-quality evidence supporting them. Such therapies should not be applied broadly to other patients with similar symptoms and should be treated with caution on a case-by-case basis. Further study on the safety and efficacy of such an approach in this patient population is needed [11].

This case report used a physiotherapy approach to pain-relieving by using TENS and cryotherapy, mat exercises for improving transitions along the basic range of motion (ROM) exercises with progressive resistance which improves strength followed by posture correction exercises such as chin tucks, and freckles exercises for improving coordination and gait. A similar study done by Olsson et al. in 2022 aimed to determine and summarize current studies on active conservative therapy of primary spinal syringomyelia and related symptoms, as well as provide therapeutic views using a hands-on strategy and used similar protocols done in this study but concluded that evaluation of factors causing symptoms and explanation for therapeutic selection was inadequate. Superior research is necessary for allowing healthcare practitioners to use evidence-based active conservative therapies [12]. By Gade et al. 2022 study concluded that a coordinated approach consisting of standard therapies and a well-planned physical therapy included improving strength and releasing the tightness of the muscle, reducing the paraesthesia and regaining the sensations, followed by reducing pain and postural correction exercises resulted in a significant improvement in the condition of the patient by significantly reducing symptoms and increasing the patient's quality of life. The efficiency of strictly scrutinized physical therapy in improving the patient's strength and alleviating symptoms after surgery [13]. The Karnofsky Performance Status (KPS) is a widely used method for determining a patient's functional status. It was proposed by David A. Karnofsky and Joseph H. Goebbels [14]. Rehabilitation programs educate patients about physical activity their benefits and the conditions they have. Pain and stiffness can be relieved with stretching and hydrotherapy. Moreover, physical therapy is said to have advantages compared to surgical intervention [15].

## Conclusions

Through physiotherapy, a more stable standard management plan was created, which resulted in a significant improvement in the patient's condition. Syringomyelia related to Arnold Chiari 1, the whole improvement was focused on reducing symptoms while enhancing the patient's quality of life. The outcome measure demonstrates a significant positive result well after physiotherapeutic intervention in functional independence measure, Berg balance scale and Karnofsky performance Index, and other areas such as pain, range of motion of cervical spine with upper limbs, strength, balance coordination, and gait. The patient can perform the basic activities of daily living (ADLs) and promote quality of life by making the patient functionally independent.
